# Delayed diagnosis of transanal prolapse of an ileo-colic intussusception in a 10-month-old infant in rural Cameroon: a case report

**DOI:** 10.1186/s13104-017-2838-8

**Published:** 2017-10-30

**Authors:** Frank-Leonel Tianyi, Benjamin Momo Kadia, Christian Akem Dimala, Valirie Ndip Agbor

**Affiliations:** 1 Kousseri Regional Hospital Annex, Kousseri, Extreme North region Cameroon; 2 Foumbot District Hospital, Foumbot, Cameroon; 3 Grace Community Health and Development Association, Kumba, Cameroon; 4 Orthopaedics Department, Southend University Hospital, Essex, UK; 5 Health and Human Development (2HD) Research Network, Douala, Cameroon; 6 Ibal Sub-divisional Hospital, Oku, North west region Cameroon

**Keywords:** Intussusception, Transanal protrusion, Rectal prolapse, Case report

## Abstract

**Background:**

Transanal protrusion of intussusception is a complication of intussusception which involves the exteriorization of the apex of the intussusceptum through the anus. However, it is rarely reported and its confusion with rectal prolapse often leads to a diagnostic delay.

**Case presentation:**

A 10-month-old female with no significant past history from a rural area in the Extreme North region of Cameroon  was referred from a local health centre to our emergency deparment for an irreducible mass. It was reported that the child had spent 5 days at home on over-the-counter medication, then 3 days at a health centre where she was being treated for a respiratory tract infection and a rectal prolapse. On arrival at our hospital, she was conscious and moderately dehydrated. Cardiopulmonary examination revealed generalized coarse crackles over both lung fields. Her abdomen was tender, with a left upper quadrant mass, absent bowel sounds and a dark anal mass. In view of these, diagnoses of bronchopneumonia, intestinal obstruction and a probable rectal prolapse were made. An exploratory laparotomy was carried out after resuscitation with per-operative findings of a prolapsed ileo- colic intussusception and a necrosed intussusceptum. The necrosed portion was resected and an end-to-end ileo-transverse anastomosis was carried out. The immediate post- operative period was uneventful, but the patient died 3 days after the surgery, from an overwhelming sepsis.

**Conclusions:**

Transanal protrusion of intussusception requires timely surgical intervention to prevent mortality. The similarity in presentation to rectal prolapse coupled with inadequate knowledge on the condition by primary healthcare personnel causes a delay in the diagnosis and an increased mortality. A high index of suspicion is essential for an early diagnosis and an improved referral system for timely and definitive treatment.

## Background

Intussusception is the invagination of a portion of the intestines within a more distal segment [1, 2], and is the most common cause of acute intestinal obstruction in children under 2 years of age [3, 4]. Transanal protrusion of intussusception (TAPI) is a rare complication of intussusception which involves the exteriorization of the apex of the intussusceptum through the anus [5]. It is an uncommon presentation of intussusception with most cases reported from low and middle-income countries of Africa and Asia [6]. The classic triad of intussusception made up of severe intermittent abdominal pain, vomiting and bloody mucoid stools, is seen in less than 1/3 of patients [7]. TAPI can present without these cardinal symptoms of intussusception [8], making its diagnosis more challenging.

Rectal prolapse is a relatively more common occurrence in childhood, and has a presentation similar to that of TAPI. Hence, most cases of TAPI are initially misdiagnosed as rectal prolapse [6]. This consequently worsens the prognosis of TAPI because of delayed intervention. Therefore, it is important to have a high index of suspicion as TAPI requires surgical management and can be associated with high morbidity and mortality.

We present the first reported case of transanal protrusion of an ileo-colic intussusception in Cameroon which was misdiagnosed as rectal prolapse leading to intestinal necrosis, extensive surgery and death from an overwhelming post-operative sepsis.

## Case presentation

A 10-month-old female with no significant past history from a rural area in the Extreme North region of Cameroon was brought to the emergency department of our primary care hospital as a referral from a local health centre where she was observed to have an irreducible necrosed anal mass. The illness started 10 days prior to arrival at our hospital with a low grade fever and cough for which her mother gave her some over-the-counter cough syrup medication. The cough persisted over the next five days, and she developed postprandial vomiting, frequent watery mucoid stools and the mother noticed a fleshy mass which will occasionally protrude from her anus. This motivated a consultation at a nearby health centre where she was admitted and treated for a respiratory tract infection and a rectal prolapse with intravenous fluids and antibiotics. Persistence of the vomiting and aggravation of the anal mass (which became larger and irreducible with a dark discoloration) prompted referral to our institution for better management.

**Fig. 1:**
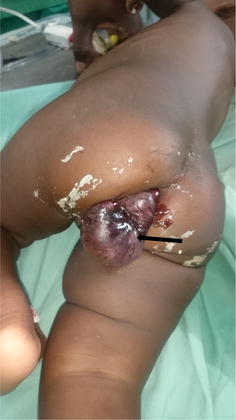
Anal protrusion of necrosed bowel

A two-day delay in arrival to our health facility after referral was noted due to financial reasons. On arrival at our hospital, 10 days after the onset of her symptoms she was conscious with a Blantyre score of 5/5, had mild flaring of the alae nasi, sunken eyes, dry lips and a poor return of skin pinch. She had a temperature of 38.9 °C, pulse of 141 beats per minute, respiratory rate of 44 breaths per minute and capillary refill time of 3s. She weighed 5.3 kgs. She had pale conjunctivae, anicteric sclerae, mild intercostal recession, and diffuse coarse crackles in both lung fields. Her abdomen was mildly distended and moved symmetrically with her breathing movements. She cried when the abdomen was palpated superficially, there was no guarding, no rigidity, and no rebound tenderness. There was an ill-defined, tender mass in the left upper quadrant, with tenderness limiting thorough examination of the mass. There were no bowels sounds. On examination of the anus, there was a dark, soft, mildly tender and irreducible mass protruding from the anus (Fig. 1). The anal margin could admit a finger next to the mass. A diagnosis of bronchopneumonia and intestinal obstruction with a necrosed anal mass was made.

A Full Blood Count revealed a moderate anaemia with a haemoglobin count of 7 g/dl, leucocytosis of 18,200 cells/mm^3^ and a thrombocytosis of 449,000 cells/mm^3^.

An analysis of her serum electrolytes was consistent with mild hyponatremia [130 mmol/l (135–145 mmol/l)], mild hypochloraemia [92 mmol/l (95–107 mmol/l)], marked hypokalaemia [2.6 mmol/l (3.6–5.1 mmol/l)] and hypomagnesaemia [12 g/l (18.2–24.3 g/l)]. Requested chest X-ray and abdominal ultrasound could not be performed because both machines were pending repairs after suffering technical faults, and the nearest hospital with these services was over 6 h away.

Initial management consisted of oxygen therapy at 2 l/min, rehydration following the WHO plan B for management of dehydration with 75 ml/kg (400 ml) of intravenous (IV) ringer’s lactate (RL) solution over the next 6 h. Antibiotherapy with ceftriaxone IV injections, 50 mg/kg (265 mg) 12 h; metronidazole IV injections, 15 mg/kg (80 mg) 12 h. Paracetamol IV injections, 15 mg/kg (80 mg) 6 h. Electrolytes, potassium chloride, 0.5 mEq/kg slowly over 2 h, 12 h. She was transfused 2 boluses of 100 cc of cross matched and compatible whole blood. A repeat of the serum electrolytes and hemoglobin concentration were within normal values.

**Fig. 2:**
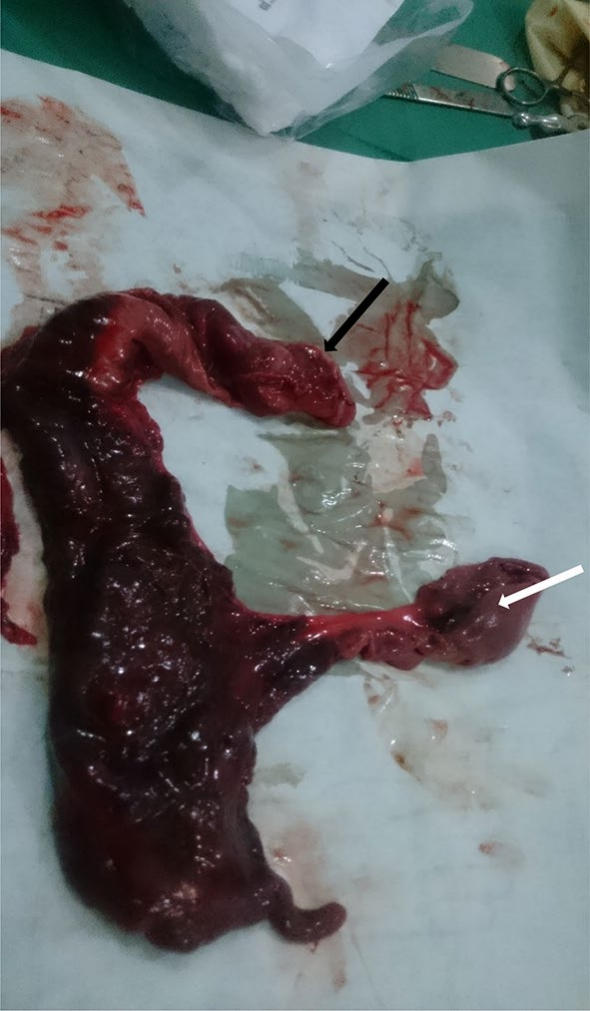
Necrosed terminal ileum, ceacum, ascending colon and proximal 1/3 of transverse colon after resection

An exploratory laparotomy was carried out after clearance by the anesthetist. This was 11 days after onset of symptoms. After the necessary pre-operative preparation, the surgery was conducted under general anaesthesia with a supra-umbilical midline incision. Intraoperative findings were an ileo-colic intussusception with the intussusceptum consisting of the terminal ileum, caecum, ascending colon, and the proximal 1/3 of the transverse colon, with the apex of the intussusceptum protruding through the anus. The prolapsed bowel was reduced with difficulties and the whole intussusceptum was observed to be necrosed after reduction. A right hemicolectomy (Fig. 2) with an end-to-end ileo-transverse anastomosis was carried out.

Post-operative evolution was stable within the first 24 h, with full recovery from anaesthesia, stable vital signs and bowel transit with passage of stools. Her condition however deteriorated within the next 48 h, as she developed a persistent high grade fever and died from an overwhelming sepsis.

## Discussion

Transanal protrusion of intussusception is defined as the invagination of an intestinal segment into the segment adjacent to it, with exteriorization of the head of the intussusceptum through the anus [5]. Most case reports on TAPI occurred in females [6, 9], which is contrary to intussusceptions with no transanal protrusions which have a male predominance [10, 11]. The average age of occurrence is 5 to 12 months [6, 8, 11]. Our patient was a 10-months old female, so she exhibited the right profile for a TAPI. A high index of suspicion of TAPI is therefore warranted when faced with children of the same age group and gender, presenting with similar symptoms. TAPI is an uncommon manifestation of childhood intussusception with anal protrusion rates ranging from 8 to 29% [8, 12, 13]. A study on 198 children with intussusception revealed 8 (4.0%) children who had a prolapsed rectal mass, with the prolapse extending beyond the anal verge in 2 (1.0%) of the children [8]. This is important because when the prolapse actually extends beyond the anal verge, as was the case in our patient, it is more likely to be misdiagnosed as a rectal prolapse.

The pathogenesis behind TAPI is not fully understood. However, proposed mechanisms include: increased intestinal peristalsis following enteritis and associated conditions, leading to rapid movement of the intussusception into the rectum [8]; anatomical defects like non-fixation of ascending and descending colons which predispose to an intussusception, with a delay in definitive treatment allowing time for transanal protrusion to occur [8].

The diagnosis of intussusception is challenging due to its varied unspecific clinical presentation, ranging from a painless intussusception to constipation, dehydration from diarrhoea and vomiting, intestinal prolapse, rectal bleeding, sepsis, shock, syncope, and altered mental status [2, 9]. The classic triad of severe intermittent abdominal pain, red currant jelly stools and vomiting occurs in less than 20% of cases [10]. Some cases of TAPI occur in the absence of cardinal signs of intussusception [8]. Also, the relatively high frequency of rectal prolapse in childhood increases the diagnostic challenge of TAPI [6]. These cause a delay in the diagnosis and referral to a surgeon for proper management. A delay in presentation for effective management is a common trend in most developing countries with patients presenting after as much as 45 days after the onset of symptoms [12]. In resource limited settings, delays in presentation are accentuated by the absence of a universal health coverage with patients having to auto-finance their cost of care. When they do get the means, their first stop is usually at primary health care centres. Here, most of the health personnel are not aware of this condition, and they delay effective management by treating for other conditions such as gastroenteritis and rectal prolapse, thereby increasing the risk of necrosis, perforation and death [14, 15]. Our case portrays the typical trend of events in resource limited settings; first the parents tried auto-medication at home, then they went to a primary health centre where the child was being managed for rectal prolapse and was only referred after the onset of complications such as necrosis and significant electrolyte and hemodynamic derangements. Lack of finances prolonged their stay at home thereby worsening the prognosis as valuable time was spent raising funds rather than seeking effective treatment. The child was operated 11 days after onset of symptoms following several failed probabilistic managements, with an unfavourable outcome.

Radiological investigations are the mainstay for the confirmation of the diagnosis of intussusception. They include Computed Tomography scannings, plain abdominal radiography, ultrasound, and contrast or air enemas which have a combined diagnostic and therapeutic effect [16]. Ultrasound scans by experienced users are very reliable in the diagnosis of intussusception [17]. However, lack of equipment and/or shortage of trained personnel limit the use of ultrasounds in resource limited settings [18, 19], posing a huge diagnostic challenge of TAPI, preoperatively. When present, lack of access to reliable maintenance and repairs have proven to be major obstacles to the use of ultrasounds [18], as was the case in this report. In resource limited settings like ours, over 90% of cases are diagnosed clinically or per operatively [2, 17]. This absence of confirmatory diagnostic work-up could cause a further delay in effective treatment and increase the advent of adverse outcomes.

Air Enema Reduction (AER) is the recommended treatment for TAPI presenting less than 48 h after onset of symptoms [8]. This practice is more common in high income countries, and is associated with decreased length of hospitalization, shorter recovery period, and decreased risk of complications associated with major abdominal surgery [20]. However, lack of specialized facilities and trained personnel, and late presentation makes an open abdominal surgery the preferred approach in resource-poor settings. Some authors propose surgical management as the only means of management for TAPI [5].

High rates of mortality are associated with: less than one year of age, delayed presentation greater than 24 h, associated peritonitis, bowel resection, and surgical site infection [20]. Our patient was 10-month-old, she presented after 11 days, had a significant portion of her bowel resected and died on post-operative day three secondary to sepsis. In line with the findings of *Chayla and co*, she had a poor prognosis from the onset, given that these conditions are usually associated with high mortality [20]. Patients satisfying one or more of these conditions should be treated with the utmost care to reduce mortality. Also, efforts should be made to raise awareness of primary health care workers in recognizing and in applying timely referral so as to decrease the morbidity, such as extensive bowel resection, and mortality associated with this disease. Diagnostic equipment should receive regular maintenance, and ultrasound use should be encouraged to aid in posing a timely diagnosis.

A high index of suspicion and an improved referral system is essential for an early diagnosis and treatment of TAPI, so as to decrease morbidity and mortality from this disease. Health policies should implement continuous medical education (CME) sessions to re-inforce the clinical capacities of primary health care workers to correctly diagnose intussusception and primary health centres should acquire and implement predefined management algorithms which favour timely referral.

## Conclusions

TAPI is a complication of intussusception which requires a prompt diagnosis and treatment to prevent mortality. The varied clinical presentation of TAPI and its close resemblance to rectal prolapse creates a diagnostic dilemma which is made worst by inadequate knowledge on the condition by primary healthcare personnel. This leads to a delay in its diagnosis and definitive management, with an increased risk for complications, intestinal necrosis, resection and death. A high index of suspicion is essential for an early diagnosis, functional ultrasound scans to assist diagnosis, improved referral system for timely and definitive treatment, and frequent CME sessions to improve diagnostic capacity of primary healthcare workers.

## Abbreviations

AER: Air Enema Reduction
CME: continuous medical education
IV: intravenous
RL: ringers lactate
TAPI: transanal protrusion of intussusception
